# Tivantinib, A c-Met Inhibitor in Clinical Trials, Is Susceptible to ABCG2-Mediated Drug Resistance

**DOI:** 10.3390/cancers12010186

**Published:** 2020-01-12

**Authors:** Zhuo-Xun Wu, Yuqi Yang, Qiu-Xu Teng, Jing-Quan Wang, Zi-Ning Lei, Jing-Qiu Wang, Sabrina Lusvarghi, Suresh V. Ambudkar, Dong-Hua Yang, Zhe-Sheng Chen

**Affiliations:** 1 Department of Pharmaceutical Sciences, College of Pharmacy and Health Sciences, St. John’s University, Queens, NY 11439, USA; zhuoxun.wu17@my.stjohns.edu (Z.-X.W.); yuqi.yang17@my.stjohns.edu (Y.Y.); qiuxu.teng15@my.stjohns.edu (Q.-X.T.); jingquan.wang16@my.stjohns.edu (J.-Q.W.); zining.lei14@my.stjohns.edu (Z.-N.L.); 18761687786@163.com (J.-Q.W.); 2 College of Chemical Engineering, Nanjing Forestry University, Nanjing 210037, China; 3 Laboratory of Cell Biology, Center for Cancer Research, National Cancer Institute, NIH, Bethesda, MD 20892, USA; sabrinal@mail.nih.gov (S.L.); ambudkar@mail.nih.gov (S.V.A.)

**Keywords:** ATP-binding cassette (ABC) transporter, tivantinib, ARQ-197, ABCG2, drug transport, multidrug resistance (MDR)

## Abstract

Tivantinib, also known as ARQ-197, is a potent non-ATP competitive selective c-Met inhibitor currently under phase 3 clinical trial evaluation for liver and lung cancers. In this study, we explored factors that may lead to tivantinib resistance, especially in regards to its interaction with ATP-binding cassette super-family G member 2 (ABCG2). ABCG2 is one of the most important members of the ATP-binding cassette (ABC) transporter family, a group of membrane proteins that play a critical role in mediating multidrug resistance (MDR) in a variety of cancers, including those of the liver and lung. Tivantinib received a high score in docking analysis, indicating a strong interaction between tivantinib and ABCG2, and an ATPase assay indicated that tivantinib stimulated ABCG2 ATPase activity in a concentration-dependent manner. An MTT assay showed that ABCG2 overexpression significantly desensitized both the cancer cells and ABCG2 transfected-HEK293 cells to tivantinib and that this drug resistance can be reversed by ABCG2 inhibitors. Furthermore, tivantinib upregulated the protein expression of ABCG2 without altering the cell surface localization of ABCG2, leading to increased resistance to substrate drugs, such as mitoxantrone. Altogether, these data demonstrate that tivantinib is a substrate of ABCG2, and, therefore, ABCG2 overexpression may decrease its therapeutic effect. Our study provides evidence that the overexpression of ABCG2 should be monitored in clinical settings as an important risk factor for tivantinib drug resistance.

## 1. Introduction

Receptor tyrosine kinases (RTKs) play a critical role in regulating cellular function. The dysregulation of certain RTKs might lead to the occurrence and progression of various cancers [1]. c-Met is a RTK that mediates a broad range of cellular signaling pathways, such as PI3K/Akt, MAPK, and FAK [2]. Strong evidence has shown that prolonged activation of c-Met is linked to tumor growth or progression. Therefore, some tyrosine kinase inhibitors (TKIs) targeting c-Met have been developed and have shown promising therapeutic effects. For example, tepotinib is under a phase 2 clinical evaluation and showed a promising effect against liver cancer (NCT02115373) and non-small cell lung cancer (NSCLC, NCT02864992) [3]. Glesatinib is another drug in a phase 2 clinical trial (NCT02954991) that investigates its therapeutic effect of NSCLC [4]. Capmatinib was also shown to be effective against NSCLC [5].

Tivantinib, also known as ARQ-197, is a non-ATP competitive c-Met inhibitor [6]. Tivantinib is being investigated as a single treatment (NCT01755767) or a combination treatment with sorafenib for hepatocellular carcinoma (HCC) [7,8]. Tivantinib is also under clinical evaluation as part of a combination treatment with erlotinib for NSCLC (NCT01244191) [9,10]. Recently, studies have shown that tivantinib is effective against cells that are c-Met independent [11]. Moreover, it has been reported that tivantinib can disrupt and inhibit microtubule polymerization [12]. The cytotoxicity of tivantinib may be related to the inhibition of GSK3α and 3β [13]. Therefore, tivantinib may have therapeutic targets other than c-Met inhibition. Generally, tivantinib is a more potent TKI compared to other c-Met inhibitors, such as tepotinib, golvantinib, and MK-2461 [14].

ATP-binding cassette (ABC) transporters are a group of membrane proteins that are closely associated with multidrug resistance (MDR). ABC drug transporters confer resistance to a broad range of chemotherapeutic agents [15,16]. They are widely expressed in various tissues, such as the small intestine and placenta, to protect organs from the efflux of toxins and xenobiotics [17]. However, overexpression of several ABC transporters in tumors is found to be an important mechanism that impedes the success of cancer treatment [18,19]. To date, P-glycoprotein (multidrug resistance protein 1/MDR1, ATP-binding cassette sub-family B member 1/ABCB1), breast cancer resistance protein (BCRP, ATP-binding cassette super-family G member 2/ABCG2, mitoxantrone resistance protein/MXR), and multidrug resistance-associated protein 1 (multidrug resistance-associated protein 1/MRP1, ATP-binding cassette super-family C member 1/ABCC1) are the prominent ABC transporters that may confer resistance to chemotherapeutic drugs [20]. ABCG2 was discovered by several groups of researchers successively. Doyle and colleagues reported the overexpression of ABCG2 in MCF-7/AdVp3000 and called it “breast cancer resistance protein” (BCRP) [21]. Later, ABCG2 cDNA was cloned from the S1-M1-80 cancer cell line and termed MXR [22]. Chemotherapeutic drugs identified as an ABCG2 substrate include mitoxantrone, irinotecan, topotecan, and doxorubicin [23]. Moreover, ABCG2 also confers resistance to some TKIs, such as nilotinib, dasatinib, and imatinib [24,25]. Previously, studies have shown that tivantinib can overcome ABCB1-mediated MDR [26,27] However, in a phase 1 study, tivantinib decreased the systemic exposure of ABCB1 substrates, indicating that it may be an inducer of ABCB1 [28].

In the present study, we report that ABCG2 overexpression can confer resistance to tivantinib, which can be reversed by ABCG2 inhibitors. This study provides evidence that ABCG2 overexpression is one of the factors contributing to the acquired resistance to tivantinib. Further, tivantinib showed a great ability to stimulate ABCG2 expression, suggesting the importance of monitoring the level of ABCG2 in cancer patients under tivantinib treatment.

## 2. Results

### 2.1. The Cytotoxicity of Tivantinib Is Attenuated in Drug-Selected or Gene Transfected ABCG2-Overexpressing Cells

**Figure 1:**
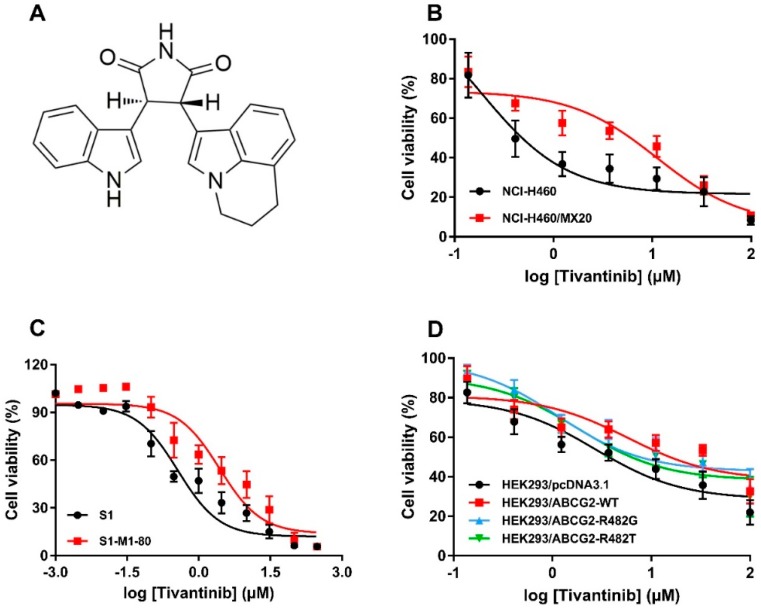
Chemical structure and cytotoxicity of tivantinib in parental and ATP-binding cassette super-family G member 2 (ABCG2)-overexpressing cells. (**A**) Chemical structure of tivantinib; (**B**) cell viability curves for NCI-H460 and NCI-H460/MX20 cells; (**C**) cell viability curves for S1 and S1-M1-80 cells; (**D**) cell viability curves for HEK293/pcDNA3.1, HEK293/ABCG2-WT, HEK293/ABCG2-R482G, and HEK293/ABCG2-R482T cells. Data are expressed as the mean ± SD from a representative of three independent experiments.

The cytotoxicity of tivantinib (the chemical structure is given in Figure 1A) in various cell lines was analyzed by MTT assay. The cell lines used in this study were NSCLC NCI-H460 and its drug-selected subline, NCI-H460/MX20, which overexpresses wild-type (WT) ABCG2 and colon cancer cell line S1, as well as its drug-selected subline, S1-M1-80, which overexpresses R482G mutant ABCG2. As shown in Figure 1B,C, ABCG2-overexpressing cells, both of the WT and mutant variants, were more resistant to tivantinib than the corresponding parental cells. To further validate the cytotoxicity of tivantinib, HEK293 cells transfected with either an empty vector, or vectors encoded with WT or mutant ABCG2 (R482G or R482T), were used. The results confirmed that both WT and mutant ABCG2-overexpressing cells were significantly less sensitive to tivantinib than parental cells, with the resistance-fold (RF) ranging from three to six-fold (Figure 1D). The IC_50_ values and RF are calculated and summarized in Table 1. These results suggest that overexpression of ABCG2 may reduce the cytotoxic effect of tivantinib. In all three variants, the WT and R482G mutant showed a higher resistance-fold (5.45-fold and 5.16-fold) than the R482T mutant (3.23-fold).

### 2.2. ABCG2 Inhibitor Sensitizes ABCG2-Overexpressing Cells to Tivantinib

To confirm that ABCG2 can confer resistance to tivantinib, reversal experiments were performed to examine whether blocking the efflux function of ABCG2 can reverse drug resistance. As shown in Table 1, 5 μM of Ko143, a potent ABCG2 inhibitor, was able to completely reverse tivantinib resistance from 4.32-fold and 3.36-fold to 1.20-fold and 1.06-fold in NCI-H460/MX20 and S1-M1-80 cells, respectively. Similarly, Ko143 was able to significantly restore the cytotoxic effect of tivantinib in ABCG2-transfected HEK293 cells. Together, these results suggest that resistance to tivantinib is associated with ABCG2 overexpression.

### 2.3. Tivantinib Stimulates the ATPase Activity of ABCG2

**Figure 2:**
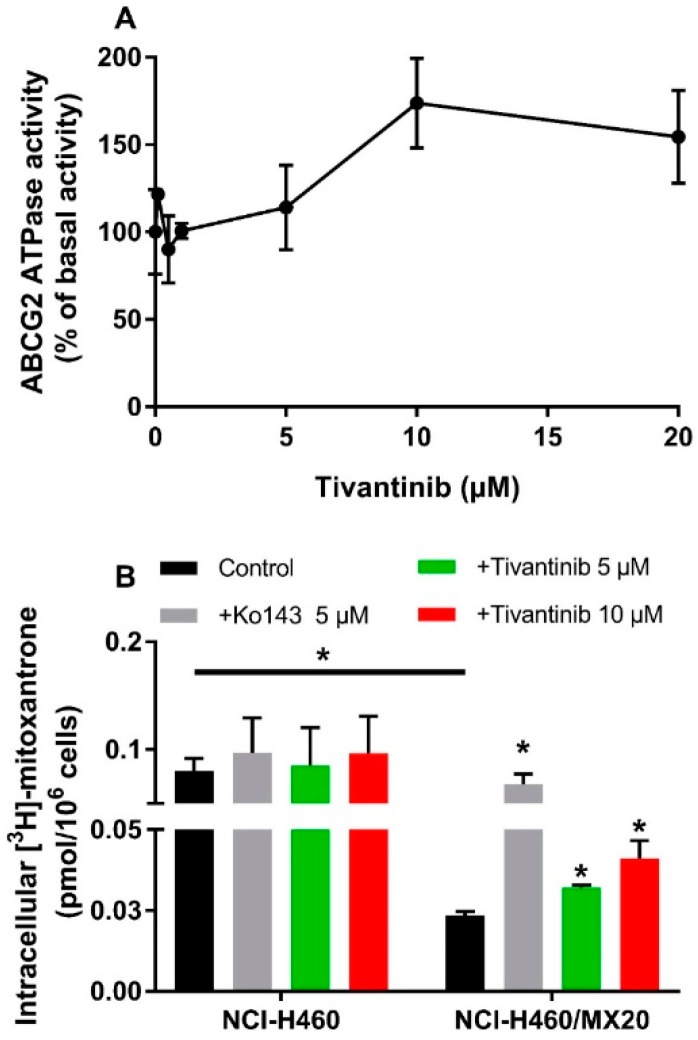
Effect of tivantinib on the ATPase activity of ABCG2 and accumulation of [^3^H]-mitoxantrone. (**A**) Tivantinib stimulates the ATPase activity of the ABCG2 transporter; (**B**) The effect of tivantinib on the intracellular accumulation of [^3^H]-mitoxantrone in NCI-H460 and NCI-H460/MX20 cells after 2 h treatment. Data are expressed as the mean ± SD from a representative of three independent experiments. * *p* < 0.05, compared with control group.

To evaluate the effect of tivantinib on ABCG2 ATPase activity, ABCG2-mediated ATP hydrolysis was measured using ABCG2 containing insect crude membranes in the presence of tivantinib (0–20 μM). Tivantinib showed concentration-dependent stimulation of ABCG2 (Figure 2A). The stimulatory effect of tivantinib reached 50% maximum stimulation at 6.76 μM and a maximum of 173.7% of basal activity. The stimulated ATPase activity indicated that tivantinib is able to interact with ABCG2, which is consistent with the above cytotoxicity results.

### 2.4. At a High-Concentration and with Short-Time Treatments, Tivantinib Increases the Intracellular Accumulation of [^3^H]-Mitoxantrone

To understand the interaction between tivantinib and ABCG2, a [^3^H]-mitoxantrone accumulation assay was conducted to evaluate the ABCG2 transporter function. It should be noted that although the concentrations of tivantinib used in this assay were much higher than those for IC_50_, the short treatment time (2 h) prevented tivantinib from impacting cell viability or ABCG2 expression. As shown in Figure 2B, 5 μM and 10 μM of tivantinib significantly increased intracellular mitoxantrone accumulation in NCI-H460/MX20 cells without affecting the accumulation in parental NCI-H460 cells. This result combined with the above results indicates that tivantinib is a substrate of ABCG2. Therefore, at high concentrations, it can compete with mitoxantrone for ABCG2 transporter activity, resulting in increased intracellular accumulation of [^3^H]-mitoxantrone.

### 2.5. In a Low-Concentration and with Long-Time Treatments, Tivantinib Decreases the Anticancer Efficacy of Substrate Drugs in ABCG2-Overexpressing Cells

**Figure 3:**
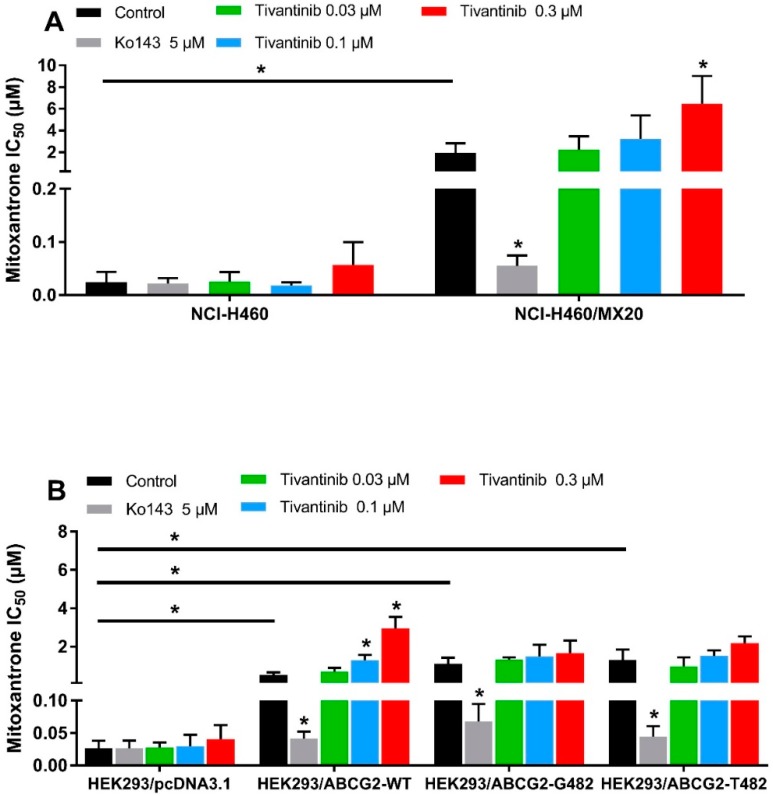
Tivantinib attenuated the cytotoxicity of mitoxantrone in parental and ABCG2-overexpressing cells. (**A**) IC_50_ values of mitoxantrone in parental NCI-H460 and drug-selected ABCG2-overexpressing NCI-H460/MX20 cells with or without treatment of tivantinib; (**B**) IC_50_ values of mitoxantrone in parental HEK293/pcDNA3.1 and transfected ABCG2-overexpressing HEK293/ABCG2-WT, HEK293/ABCG2-R482G, and HEK293/ABCG2-R482T cells with or without treatment of tivantinib. Data are expressed as the mean ± SD from a representative of three independent experiments. * *p* < 0.05, compared with the control group.

It is known that some ABCG2 reversal agents are substrates of ABCG2 and work by competing with other substrate drugs for ABCG2 activity, leading to the increased intracellular accumulation of substrate drugs. The accumulation assay indicated that tivantinib, at high concentrations and short exposure times, works like these other reversal agents by competing with mitoxantrone for drug efflux. However, to stimulate conditions more similar to a clinical setting, we wanted to examine, using an MTT assay, whether tivantinib can reverse ABCG2-mediated drug resistance at low-toxic concentrations after 72 h of treatment. To avoid the additive toxic effect of tivantinib and mitoxantrone, low concentrations (0.01–0.3 μM) of tivantinib were selected for the reversal study. NCI-H460/MX20 cancer cells and transfected HEK293 cells were used to carry out the reversal experiments. Surprisingly, rather than reversing the drug resistance, tivantinib enhanced resistance to mitoxantrone in resistant cells in a concentration-dependent manner, as shown in Figure 3A. Tivantinib, at 0.3 μM, significantly increased the IC_50_ values of mitoxantrone in NCI-H460/MX20 cells without affecting those in the parental cells. Tivantinib showed similar effects in the HEK293/ABCG2-WT cells but not for the cells expressing the mutant R482G or R482T (Figure 3B). In contrast, Ko143 was used as a positive inhibitor and significantly reversed mitoxantrone resistance. Furthermore, tivantinib combined with the non-substrate drug cisplatin did not affect the cytotoxicity of cisplatin (see Table S1). These results indicate that tivantinib may enhance ABCG2-mediated MDR.

### 2.6. Tivantinib Increases the Expression of ABCG2 Without Affecting Cell Surface Localization

**Figure 4:**
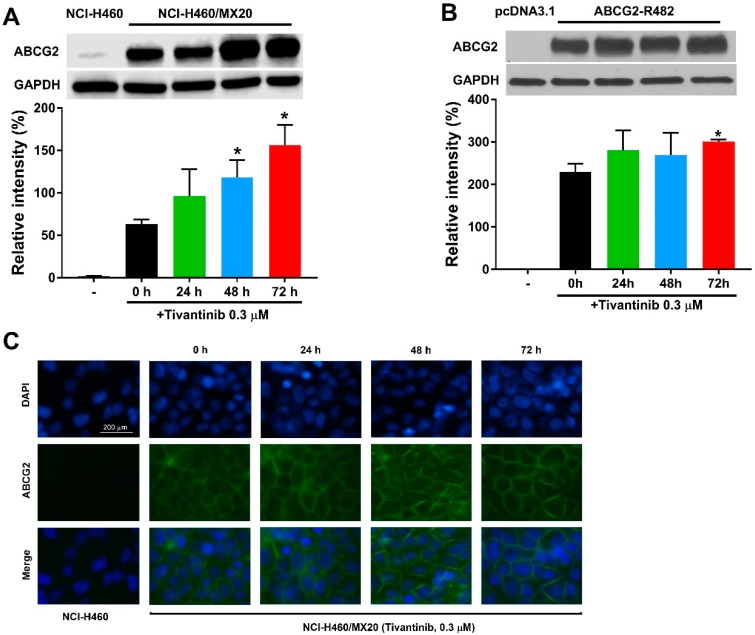
Tivantinib increases the protein expression in cells overexpressing ABCG2 without affecting the ability of ABCG2 to localize on the cell surface. (**A**) The effect of tivantinib on the protein expression of ABCG2 in NCI-H460/MX20 cells treated with 0.3 μM of tivantinib for 0, 24, 48, and 72 h. (**B**) The effect of tivantinib on the protein expression of ABCG2 in HEK/293-ABCG2-WT cells treated with 0.3 μM of tivantinib for 0, 24, 48, and 72 h. (**C**) Cell surface localization of ABCG2 expression in NCI-H460/MX20 cells incubated with 0.3 μM of tivantinib for 0, 24, 48, and 72 h. Data are expressed as the mean ± SD from a representative of three independent experiments. * *p* < 0.05, compared with the control group. scale bar: 200 μm.

Since the upregulation of ABCG2 protein expression may account for the increased resistance in tivantinib-treated cells, we postulated that ABCG2 protein expression could be enhanced by tivantinib. Thus, we examined the effect of tivantinib on ABCG2 protein expression in both drug-selected cancer cells and gene transfected cells using Western blotting and immunofluorescent staining. As shown in Figure 4A,B, and Figure S2, 0.3 μM of tivantinib was able to upregulate the protein expression in resistant NCI-H460/MX20 cells and HEK293/ABCG2-WT cells. These results, at least in part, can be used to explain the underlying mechanism for the increased resistance to mitoxantrone, an ABCG2 substrate drug. Of note, vehicle (dimethyl sulfoxide, DMSO) treatment did not affect to the ABCG2 expression level in either the parental or resistant cells, as shown in Figure S1. Furthermore, the immunofluorescence assay suggested that tivantinib did not change the cell surface localization of ABCG2, as shown in Figure 4C.

### 2.7. Low Concentrations of Tivantinib Decrease the Intracellular Accumulation of [^3^H]-Mitoxantrone after 72 h of Treatment

**Figure 5:**
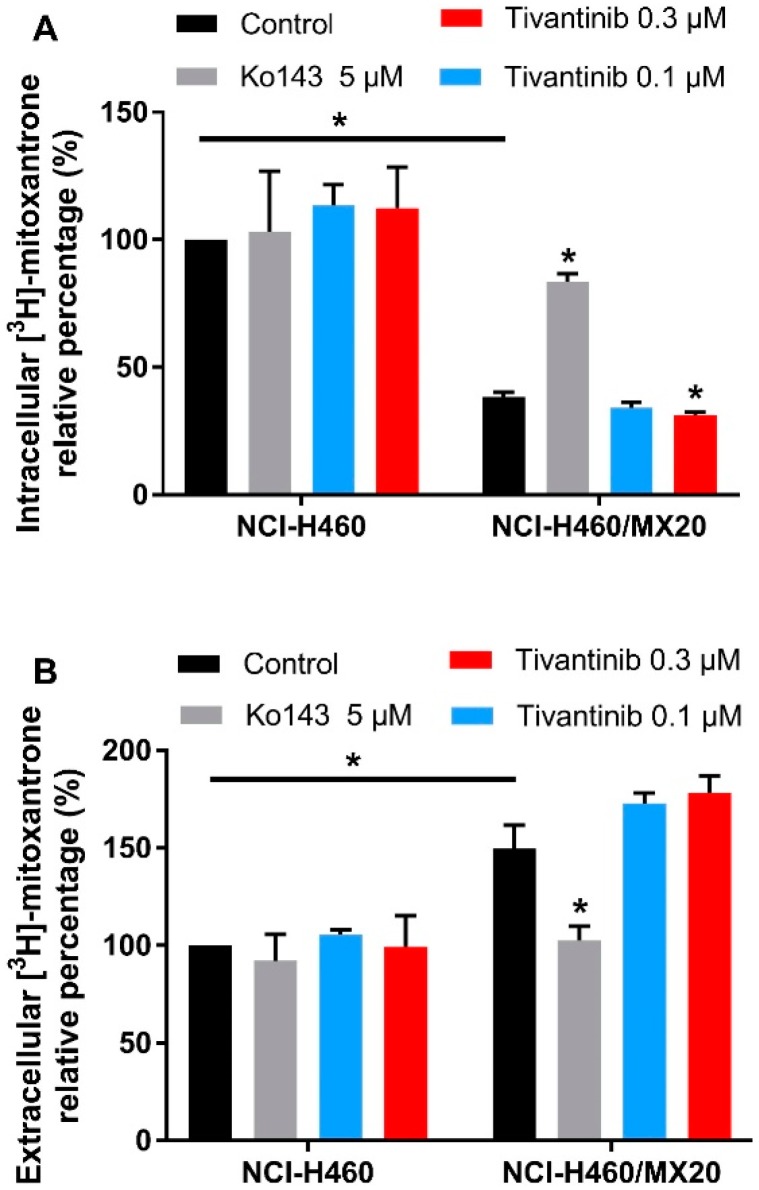
The effect of tivantinib on the accumulation of [^3^H]-mitoxantrone after 72 h treatment. (**A**) The effect of tivantinib on the intracellular accumulation of [^3^H]-mitoxantrone in NCI-H460 and NCI-H460/MX20 cells after 72 h treatment. (**B**) The relative percentage of [^3^H]-mitoxantrone in the medium after 72 h tivantinib treatment in NCI-H460 and NCI-H460/MX20 cells. The percentages were calculated as the treatment groups divided by the parental control group. Data are expressed as the mean ± SD from a representative of three independent experiments. * *p* < 0.05, compared with the control group.

The above cytotoxicity and Western blotting results indicate that tivantinib can upregulate ABCG2 expression and increase the IC_50_ values of the substrate drug in drug-resistant cells after a low-dose and 72 h of treatment. We performed a [^3^H]-mitoxantrone accumulation assay to examine whether more mitoxantrone would be pumped out of the cells after tivantinib treatment. The cells were incubated with tivantinib and [^3^H]-mitoxantrone for 72 h, and the medium and cell pellets were collected to determine the amount of [^3^H]-mitoxantrone. As shown in Figure 5A, tivantinib was able to decrease the intracellular concentration of mitoxantrone. In contrast, the ABCG2 inhibitor Ko143 significantly increased the intracellular accumulation of mitoxantrone in drug resistant cells. We also measured the remaining mitoxantrone in the medium (Figure 5B). The data showed a significantly decreased extracellular amount of mitoxantrone in the Ko143 treatment group, while the tivantinib treatment samples showed a slight increase in the amount of extracellular mitoxantrone. Moreover, treatment with either tivantinib or Ko143 did not affect the mitoxantrone accumulation in parental NCI-H460 cells. These data further confirm that tivantinib can stimulate the protein expression of ABCG2, leading to a greater amount of substrate drug being pumped from the drug resistant cells.

### 2.8. Analysis of Tivantinib and ABCG2 Interaction by Molecular Docking

**Figure 6:**
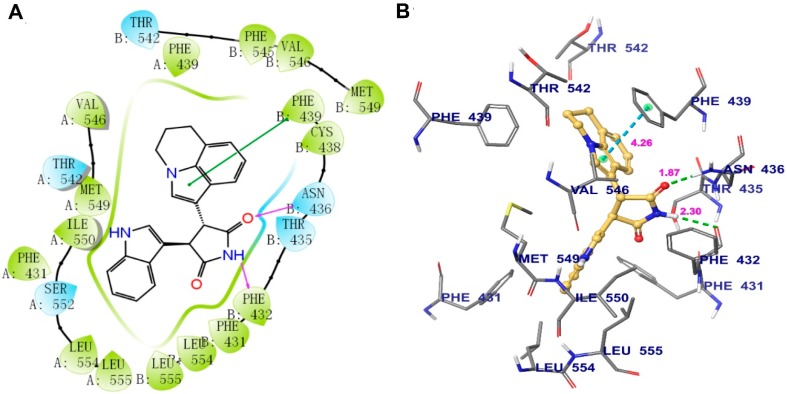
The binding mode of tivantinib to the human ABCG2 model, as predicted by induced-fit docking. (**A**) A 2D schematic diagram of the ligand–receptor interaction between tivantinib and the human ABCG2 model. Amino acids within 4 Å are indicated as colored bubbles, polar residues are depicted in blue, and hydrophobic residues are depicted in green. Purple arrows denote H-bonds and green lines denote π–π stacking aromatic interactions. (**B**) The docked conformation of tivantinib (ball and rod model) is shown within the ABCG2 drug-binding cavity, with the atoms colored as follows: carbon, yellow; hydrogen, white; oxygen, red; nitrogen, blue. Important amino acid residues are described (rods model) with the same color scheme as above for all atoms but with carbon atoms in gray. Dotted green lines represent hydrogen-bonding interactions, while dotted azure lines represent π–π stacking interactions. The values of the correlation distances are indicated in Å.

Induced-fit docking (IFD) was performed to predict the interaction between tivantinib and human ABCG2. The highest Glide score of tivantinib was -14.238 kcal/mol, which indicates the binding free energy of the ligand. The optimal scoring pose of tivantinib coincided with the ligand-binding cavity in the transmembrane region, which is composed of residues PHE431, PHE439, THR542, VAL546, MET549, ILE550, SER552, LEU554, and LEU555 in protein chain A and PHE431’(prime), PHE432, THR435, ASN436, CYS438, PHE439, THR542, PHE549, VAL546, MET549, LEU554, and LEU555 in protein chain B (Figure 6A). As shown in Figure 6B, two hydrogen bonds were formed with protein chain B: one between the pyrrolidinedione group of tivantinib and ASN436 and another between the same pyrrolidinedione group and PHE432. In addition, π–π stacking interactions were observed between the pyrroline ring of tivantinib and the side chain of PHE439.

## 3. Discussion

The MDR-linked ABCG2 transporter remains one of the major obstacles for cancer chemotherapy. The overexpression of ABCG2 has been reported in leukemia and in solid tumors, such as NSCLC and liver cancer [29]. Many conventional chemotherapeutic agents, such as mitoxantrone, doxorubicin, topotecan, and irinotecan have been identified as substrates of ABCG2. Some TKIs, such as imatinib and gefitinib, were also reported as substrates of ABCG2 [30]. These substrate drugs have attenuated the antineoplastic efficacy in ABCG2-overexpressing cancer cells. Therefore, identifying drugs that are substrates of ABC drug-transporters can provide important indications for treatment strategies.

Multiple c-Met inhibitors are currently under clinical investigation for hepatocellular carcinoma and some inhibitors, such as tepotinib, tivantinib, and capmatinib, show a promising therapeutic effect [31]. Tivantinib is one of the most well-developed c-Met inhibitors, with multiple ongoing clinical trials. Therefore, it is critical to identify the potential factors that may lead to acquired resistance to tivantinib. Although tivantinib was firstly described not to be a substrate of ABCB1, ABCG2, or ABCC1, clinical studies have shown that tivantinib may be a weak substrate and inducer of ABCB1 and have the potential to reduce ABCB1 substrate drug exposure [26,27,28].

In this study, we confirmed that ABCG2 can confer resistance to tivantinib, which may challenge its clinical therapeutic effect. We first examined the cytotoxicity of tivantinib in parental and ABCG2-mediated MDR cell lines. All the ABCG2-overexpressing cells showed notable resistance to tivantinib treatment compared to those of the corresponding parental cells. Furthermore, it was documented that a mutation at the position 482 of the ABCG2 protein can result in a distinct substrate-binding and efflux profile [32,33]. Our results showed that both WT and mutant ABCG2-overexpressing cells were resistant to tivantinib., Therefore, the R482 mutation has little effect on tivantinib resistance. Tivantinib resistance was also found in ABCG2-transfected HEK293 cells, in which ABCG2 overexpression is the solo contributor to MDR. Hence, we hypothesized that tivantinib could be a universal substrate of ABCG2. More importantly, the acquired resistance to tivantinib can be re-sensitized in the presence of an ABCG2 inhibitor, indicating that the overexpression of ABCG2 is the mechanism of tivantinib resistance. Since ABC transporters utilize energy from ATP hydrolysis to pump out substrate drugs, an ATPase assay was performed to determine whether tivantinib can stimulate ABCG2 ATPase activity. In the presence of transported substrates, such as imatinib, nilotinib, the ATPase activity of the transporter will increase [25]. Therefore, ATPase activity provides direct evidence that the drug is a substrate of ABCG2. Consistently, we observed a significant concentration-dependent stimulatory effect of ABCG2 ATPase activity, which demonstrated that tivantinib is a substrate of ABCG2.

It is known that some ABCG2 inhibitors, such as Ko143 and FTC, can restore the chemotherapeutic effect of anticancer agents by inhibiting ATPase function [34,35]. Mechanistic studies suggested that some substrates can function as competitive inhibitors that help overcome ABC transporter-mediated MDR [30,36,37]. Therefore, we investigated whether tivantinib, as a substrate of ABCG2, can compete with other chemotherapeutic drugs at the substrate binding site of the ABCG2 transporter and thus reverse drug resistance. The [^3^H]-mitoxantrone accumulation assay showed that, after 2 h of co-incubation of mitoxantrone with high concentrations (5 or 10 μM) of tivantinib, an increased intracellular accumulation of mitoxantrone was detected in the drug-resistant cells but not in the corresponding drug-sensitive cells. This effect might be the result of a high concentration of tivantinib being able to competitively inhibit the efflux of mitoxantrone and in turn increasing intracellular mitoxantrone accumulation. Since the cells were incubated with tivantinib for a short time, tivantinib was unlikely to alter other cellular functions, suggesting competitive inhibition as the major factor leading to increased accumulation. These data further confirm that tivantinib is a substrate of ABCG2.

Subsequently, the ability of tivantinib to reverse drug resistance at low toxic concentrations was evaluated. Due to the relatively high toxicity of tivantinib, the concentrations (5 or 10 μM) used in the aforementioned accumulation assay would be too toxic and may not represent an effect in clinical settings. Instead, non-toxic concentrations (0.03–0.3 μM) were selected to circumvent additive toxicity. At low concentrations, tivantinib failed to sensitize the ABCG2-overexpressing MDR cells to mitoxantrone, a conventional anti-neoplastic drug. However, it was observed that 0.3 μM of tivantinib enhanced mitoxantrone resistance in ABCG2-overexpressing drug-selected cells without affecting mitoxantrone sensitivity in the parental cell line. Notably, a similar effect was observed in tivantinib-treated ABCG2-transfected HEK293 cells. Furthermore, treatment with tivantinib did not affect the cytotoxicity of non-substrate drug cisplatin, suggesting that the effect may be specific to ABCG2. Because stimulation of the protein expression of ABCG2 could be a possible underlying mechanism, Western blotting was conducted to further evaluate the mechanism of action. The result suggested that, within 72 h, a low dose of tivantinib was able to upregulate ABCG2 protein levels in drug resistant cells, which confirmed ABCG2 protein upregulation as a reason for increased drug resistance. It has been reported that solvent DMSO may affect gene expression or cellular process [38,39]. In Figure S1, we ruled out the possibility that DMSO treatment can upregulate ABCG2 expression, indicating that the upregulation of ABCG2 is due solely to tivantinib treatment. However, a detailed mechanism of this upregulation should be investigated further. Moreover, the immunofluorescent results showed that tivantinib did not affect the cell surface localization of the ABCG2 transporter. Combined with the results of Western blotting, we postulate that not only was the ABCG2 protein upregulated but also continued to be localized at the cell surface, allowing a more functionable ABCG2 transporter to pump out its substrates. To further evaluate our assumption, a 72 h accumulation assay was performed using the same concentrations as in the reversal studies, and the intracellular and extracellular amounts of [^3^H]-mitoxantrone were quantified. We hypothesized that if tivantinib can upregulate ABCG2 expression and make the cells more resistant to both tivantinib and mitoxantrone, a trend of decreased intracellular mitoxantrone would be detected after 72 h of treatment. The results showed that tivantinib treatment decreased intracellular accumulation and increased levels of extracellular mitoxantrone in drug-resistant cells. Moreover, this effect was not observed in the parental cells, which is consistent with the cytotoxicity results. Previous studies have shown that tivantinib can overcome ABCB1-mediated MDR but may lead to ABCB1 induction. Therefore, tivantinib treatment may result in a decreased exposure to other substrate drugs, such as vincristine and digoxin [28]. Our study provides evidence that tivantinib is not only a substrate of ABCG2 but also induces the expression of ABCG2. Therefore, tivantinib at high concentrations has the ability to competitively inhibit mitoxantrone efflux, but tivantinib at low concentrations may also lead to ABCG2 upregulation, which paradoxically increases mitoxantrone efflux. We also noticed that although tivantinib is a substrate of all three variants of ABCG2, the ABCG2 upregulation effect was observed only in WT ABCG2 but not the mutant R482G and R482T variants. It is worthwhile to study whether a similar effect would be observed in mutant variants by prolonging the treatment time or increasing the treatment concentration. It is also possible that this effect may be specific to WT ABCG2 due to the allosteric effect. Further studies are needed to investigate whether other mechanisms are involved in this interaction.

Furthermore, a computational molecular docking analysis was performed to predict the interactions between tivantinib and the ABCG2 transporter. The IFD analysis simulated the molecular interactions between tivantinib and the human ABCG2 drug-binding pocket. The results predicted a high binding affinity between tivantinib and the human ABCG2 drug-binding pocket, with a score of −14.238 kcal/mol. In order to verify the possibility that tivantinib may be a human ABCG2 substrate, we further analyzed the known ABCG2 substrates mitoxantrone and topotecan via IFD with the same parameters. The optimal scores of mitoxantrone and topotecan were −12.350 kcal/mol and −13.337 kcal/mol, respectively, which suggests that the affinity of tivantinib with ABCG2 transporter may be comparable to other well-established substrates. These results indicate that tivantinib is a substrate of human ABCG2.

## 4. Materials and Methods

### 4.1. Reagents

Tivantinib (ARQ-197) was purchased from ChemieTek (Indianapolis, IN, USA). Dulbecco’s modified Eagle’s Medium (DMEM), trypsin EDTA 1X, penicillin/streptomycin, and fetal bovine serum (FBS) were purchased from Corning Incorporated (Corning, NY, USA). Phosphate buffer saline (PBS) and Bovine Serum Albumin (BSA) were obtained from VWR chemicals, LLC (Solon, OH, USA). The anti-BCRP antibody, clone BXP-21 (catalog number MAB4146, Lot number 3026758), was purchased from Millipore (Billerica, MA, USA). The GAPDH loading control monoclonal antibody (GA1R) (catalog number MA5-15738, Lot number SA247966) and Alexa Fluor 488 conjugated goat anti-mouse IgG cross-adsorbed secondary antibody were purchased from Thermo Fisher Scientific Inc (Rockford, IL, USA). The Horseradish peroxidase (HRP)-conjugated rabbit anti-mouse IgG secondary antibody (catalog number 7076S, Lot number 32) was purchased from Cell Signaling Technology Inc (Danvers, MA, USA). [^3^H]-mitoxantrone (2.5 Ci/mmol) was purchased from Moravek Biochemicals, Inc. (Brea, CA, USA). Ko143 was purchased from Enzo Life Sciences (Farmingdale, NY, USA). Cisplatin, mitoxantrone, methylthiazolyldiphenyl-tetrazolium bromide (MTT), dimethyl sulfoxide (DMSO), paraformaldehyde, triton X-100, and 4′,6-diamidino-2-phenylindole (DAPI), and all other chemicals were purchased from Sigma Chemical Co (St. Louis, MO, USA).

### 4.2. Cell Lines and Cell Culture

The ABCG2-overexpressing resistant cell line NCI-H460/MX20 was established by introducing step-wise increased doses of mitoxantrone to parental human NSCLC NCI-H460 cells, which were maintained in a medium with 20 nM of mitoxantrone [40]. The human colon carcinoma cell line S1 and its mitoxantrone-selected ABCG2 overexpressing S1-M1-80 cells were selected through a similar process and were maintained in a medium containing 80 μM of mitoxantrone [22]. HEK293/pcDNA3.1 and HEK293/ABCG2 were transfected with either an empty vector pcDNA3.1 or a pcDNA3.1 vector containing a full length ABCG2. Transfected cells were selected with a medium containing G418 (2 mg/mL). All cells were cultured in a DMEM medium containing 10% (*v/v*) fetal bovine serum and 1% (*v/v*) penicillin/streptomycin and maintained at 37 °C in an incubator containing 5% CO_2_. They were then grown as an adherent monolayer. The drug-resistant cells were grown in a drug-free culture media for more than 14 days before the experiment.

### 4.3. MTT Assay for Cell Viability

An MTT assay was performed to determine the cytotoxicity of tivantinib and other chemotherapeutic agents in the cancer cells, as previously described [41]. Briefly, 5000 cells were seeded evenly into 96-well plates and incubated for 24 h. On the second day, serial dilutions of the substrate drugs were added to the designated wells with or without the presence of reversal agents. The cells were then incubated for 72 h. On the last day, an MTT solution was added and further incubated for 4 h at 37 °C. After incubation, the supernatant was discarded, and 100 μL of DMSO was added to dissolve the formazan crystal. The light absorbance was measured at 570 nm using an accuSkanTM GO UV/Vis Microplate Spectrophotometer (Fisher Sci., Fair Lawn, NJ, USA). The IC_50_ value and resistance-fold (RF) were calculated as described previously [42].

### 4.4. [^3^H]-Mitoxantrone Accumulation Assay

Cells (1 × 10^5^) were seeded evenly into 24-well plates and incubated for 24 h at 37 °C prior to the experiment. Reversal agents were added to the designated wells and incubated for 2 h. Then, the cells were washed with PBS and incubated in a medium containing 20 nM of [^3^H]-mitoxantrone and tivantinib or Ko143 for the designated incubation time. Subsequently, the cells were collected and transferred into scintillation vials, and scintillation fluid was added. The radioactivity reading was measured using a Packard TRICARB 1900CA liquid scintillation analyzer (Packard Instrument, Downers Grove, IL, USA).

### 4.5. Western Blotting Analysis

Cells were treated with 0.3 μM tivantinib for different time periods (0, 24, 48, and 72 h), and then the lysates were collected. To ensure equal protein loading, the protein concentrations of the cell lysates were determined using a Pierce™ BCA Protein Assay Kit (Thermo Scientific, Rockford, IL, USA). Protein samples were prepared and subjected to electrophoresis on a 10% acrylamide SDS gel and then transferred onto PVDF membranes. In order to minimize non-specific binding, the PVDF membranes were blocked with 5% non-fat milk. After blocking, the membranes were immunoblotted with primary antibodies against ABCG2 or GAPDH overnight at 4°C. On the second day, the membranes were washed with a TBST buffer followed by incubation with HRP-conjugated anti-mouse antibody (1:1000) for 2 h at room temperature. After incubation, the protein bands were visualized using a Pierce™ ECL Western Blotting Substrate (Thermo Scientific, Rockford, IL, USA). The results were quantified and analyzed using the ImageJ software (NIH, Bethesda, MD, USA).

### 4.6. Immunofluorescence Assay

NCI-H460 and NCI-H460/MX20 cells (1 × 10^5^) were seeded evenly into 24-well plates and incubated overnight. The cells were treated with or without 0.3 μM of tivantinib for different time points (0, 24, 48, 72 h). After 3 days treatment, the cells were fixed in 4% formaldehyde, permeabilized by 0.25% Triton X-100, and blocked with 6% BSA (dissolved in PBS). Subsequently, the cells were immunoblotted with a monoclonal antibody against ABCG2 (1:1000) overnight at 4 °C. The following day, the cells were incubated with an Alexa Fluor 488 conjugated anti-mouse IgG secondary antibody solution. The cells were then incubated with a DAPI solution at 37 °C for 15 min. Images were taken with a Nikon TE-2000S fluorescence microscope (Nikon Instruments Inc., Melville, NY, USA).

### 4.7. ATPase Assay

The vanadate-sensitive ATPase activity of ABCG2 was measured as previously described [43]. Insect cell membrane vesicles containing ABCG2 (10 µg total protein) were incubated in an assay buffer containing 50 mM MES (pH 6.8), 50 mM KCl, 5 mM sodium azide, 2 mM EGTA, 2 mM DTT, 1 mM ouabain, and 10 mM MgCl_2,_ with or without 300 mM sodium orthovanadate. Then, variable concentrations of tivantinib (0–20 µM final concentration) were incubated with the membrane vesicles for 3 min at 37 °C. The ATP hydrolysis was initialized via the addition of 5 mM ATP. After incubation at 37° C for 20 min, the reaction was terminated by the addition of an SDS solution at a 2.5% final concentration. Inorganic phosphate (P_i_) was quantified by a colorimetric method, as previously described [44,45].

### 4.8. Molecular Docking Analysis Using the Human ABCG2 Model

The cryo-EM structure of the human ABCG2 model (PDB code: 6ETI) was prepared, and the docking grid was confined, at the drug-binding cavity by selecting residues with important interactions [46]. The protocol of molecular docking was as previously described [47] and performed using the Maestro v11.1 software (Schrödinger, LLC, New York, NY, USA). After preparing the ligands with a low-energy pose, all possible structures were subjected to the prepared protein model through Glide XP (extra precision) docking analysis (Schrödinger, LLC, New York, NY, USA). The top-score results of Glide XP were then used to conduct induced-fit docking (IFD), and the highest-scoring result was used for the final graphic analysis. All computer analyses were performed on a 6-core Intel Xeon Processor with Mac OS. The results of the docking scores were calculated and demonstrated as kcal/mol.

### 4.9. Statistical Analysis

All experiments were performed and repeated at least three times. All data were expressed as the mean ± SD and analyzed using a one-way ANOVA. Results were considered to be of statistical significance if *p* < 0.05.

## 5. Conclusions

Our study indicates that the overexpression of ABCG2 is a major factor leading to tivantinib resistance. Tivantinib is a substrate of ABCG2 that may result in attenuated anticancer effects in ABCG2-overexpressing cells; these effects can be antagonized by co-treatment with a known ABCG2 inhibitor. In addition, tivantinib induced ABCG2 upregulation, which may affect the pharmacokinetics of other ABCG2 substrate drugs, such as mitoxantrone. The ABCG2-mediated resistance to tivantinib may have clinical implications for patients using tivantinib for cancer treatment.

## Figures and Tables

**Table 1:** The cytotoxicity of tivantinib in cells overexpressing ABCG2.

Cell Line	IC_50_ ± SD ^a^ μM, (Resistance-fold ^b^)			
	Mitoxantrone	Mitoxantrone + Ko143 5 μM	Tivantinib	Tivantinib + Ko143 5 μM
NCI-H460	0.025 ± 0.016 (1.00)	0.022 ± 0.007 (0.89)	0.764 ± 0.291 (1.00)	0.640 ± 0.394 (0.84)
NCI-H460/MX20	1.939 ± 0.759 * (78.56)	0.055 ± 0.014 (2.22)	3.295 ± 0.375 * (4.32)	0.914 ± 0.247 (1.20)
S1	0.033 ± 0.015 (1.00)	0.026 ± 0.011 (0.79)	0.712 ± 0.038 (1.00)	0.643 ± 0.136 (0.90)
S1-M1-80	4.563 ± 1.513 * (140.04)	0.138 ± 0.044 (4.22)	2.391 ± 0.442 * (3.36)	0.756 ± 0.193 (1.06)
HEK293/pcDNA3.1	0.024 ± 0.010 (1.00)	0.026 ± 0.011 (1.07)	3.333 ± 1.702 (1.00)	4.404 ± 1.549 (1.32)
HEK293/ABCG2-WT	0.521 ± 0.123 * (21.29)	0.042 ± 0.009 (1.70)	18.151 ± 3.860 * (5.45)	7.347 ± 2.755 * (2.20)
HEK293/ABCG2-R482G	1.099 ± 0.301 * (44.87)	0.119 ± 0.023 (4.85)	17.216 ± 7.144 * (5.16)	4.640 ± 1.587 (1.39)
HEK293/ABCG2-R482T	1.082 ± 0.174 * (44.20)	0.033 ± 0.017 (1.35)	10.752 ± 2.168 * (3.23)	4.328 ± 1.661 (1.30)

^a^ The half maximal inhibitory concentration (IC_50_) values are represented as mean ± SD of at least three independent experiments performed in triplicate. ^b^ Rf: Resistance-fold was calculated by dividing the IC_50_ values of ABC transporter overexpressing cells by the IC_50_ of the corresponding parental cells in the presence of mitoxantrone or tivantinib and in the absence of Ko143. * *p* < 0.05.

## Supplementary Materials

The following are available online at https://www.mdpi.com/2072-6694/12/1/186/s1, Figure S1. The protein expression in cells overexpressing ABCG2, Figure S2. Western blot for Figure 4, Table S1. The effect of tivantinib on cytotoxicity of cisplatin in cells overexpressing ABCG2.
