# Supplementary material for: Tivantinib, A c-Met Inhibitor in Clinical Trials, Is Susceptible to ABCG2-Mediated Drug Resistance
Source: Cancers (Basel). 2020 Jan 12;12(1):186. doi: 10.3390/cancers12010186 (PMC7017082; doi:10.3390/cancers12010186)
Supplement: Supplementary file 1 [file cancers-12-00186-s001.pdf]

Supplementary Materials: Tivantinib, A c-Met Inhibitor in Clinical Trials, Is Susceptible to ABCG2-Mediated Drug Resistance

Zhuo-Xun Wu, Yuqi Yang, Qiu-Xu Teng, Jing-Quan Wang, Zi-Ning Lei, Jing-Qiu Wang, Sabrina Lusvarghi, Suresh V. Ambudkar, Dong-Hua Yang and Zhe-Sheng Chen

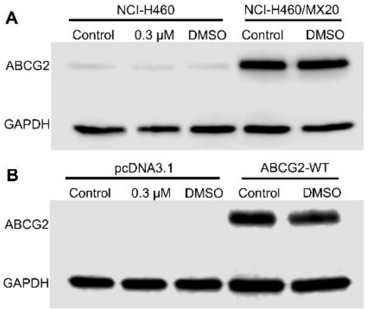

Figure S1. The protein expression in cells overexpressing ABCG2 (**A**) The protein expression of ABCG2 in NCI-H460 and NCI-H460/MX20 cells treated with 0.3 μM of tivantinib or 0.003% (*v/v*) DMSO for 72 h. (**B**) The protein expression of ABCG2 in HEK293/pcDNA3.1 and HEK293/ABCG2-WT cells treated with 0.3 μM of tivantinib or DMSO for 72 h.

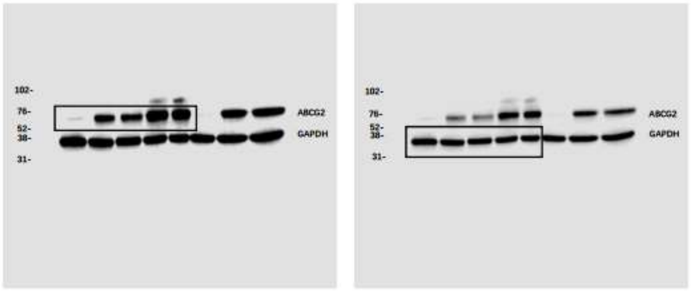

IntDen	H460	MX20-0 h	MX20-24 h	MX20-48 h	MX20-72 h
ABCG2	7217	265585	261276	438594	372669
GAPDH	503135	445402	423633	383888	323347
Relative intensity (%)	1.4344	59.6282	61.6751	114.2505	115.2536

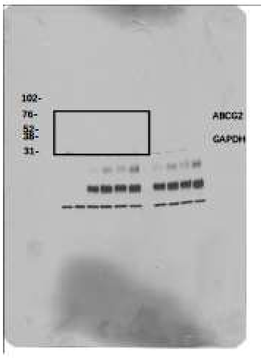

IntDen	pcDNA3.1	ABCG2-0 h	ABCG2-24 h	ABCG2-48 h	ABCG2-72 h
ABCG2	900	313281	381655	366244	399393
GAPDH	142921	145188	154545	157643	134059
Relative intensity (%)	0.629719	215.7761	246.954	232.3249	297.9233

Figure S2. Western blot for Figure 4.

Table S1. The effect of tivantinib on cytotoxicity of cisplatin in cells overexpressing ABCG2.

Cell Line	IC_{50} ± SD[a] μM, (Resistance-fold[b])		
	**Cisplatin**	**Cisplatin + Ko143 5 μM**	**Cisplatin + Tivantinib 0.3 μM**
NCI-H460	3.900 ± 0.664 (1.00)	4.037 ±0.503 (1.04)	5.125 ± 0.329 (1.31)
NCI-H460/MX20	3.991 ± 0.187 (1.02)	4.750 ± 0.213 (1.22)	3.894 ± 0.377 (1.00)
HEK293/pcDNA3.1	6.482 ± 0.640 (1.00)	8.306 ± 1.238 (1.28)	6.820 ± 0.521 (1.05)
HEK293/ABCG2-WT	7.491 ± 1.022 (1.16)	7.094 ± 0.240 (1.70)	8.603 ± 1.321 (1.33)
HEK293/ABCG2-R482G	6.672 ± 1.159 (1.03)	8.335 ± 0.565 (1.29)	6.860 ± 0.223 (1.06)
HEK293/ABCG2-R482T	7.462 ± 1.192 (1.15)	9.098 ± 0.648 (1.40)	8.898 ± 0.280 (1.37)

[a] IC_{50} values are represented as mean ± SD of at least three independent experiments performed in triplicate. [b] Rf: Resistance-fold was calculated by dividing the IC_{50} values of ABC transporter overexpressing cells by the IC_{50} of the corresponding parental cells in the presence of cisplatin and in the absence of Ko143 or tivantinib. * $p < 0.05$ versus the control group.

© 2020 by the authors. Licensee MDPI, Basel, Switzerland. This article is an open access article distributed under the terms and conditions of the Creative Commons Attribution (CC BY) license (http://creativecommons.org/licenses/by/4.0/).
